# The Effect of Combining the COVID-19 Vaccine with the Seasonal Influenza Vaccine on Reducing COVID-19 Vaccine Rejection Among Libyans

**DOI:** 10.1007/s44197-023-00107-2

**Published:** 2023-05-12

**Authors:** Ramy Mohamed Ghazy, Malik Sallam, Fatimah Saed Alabd Abdullah, Mai Hussein, Mohamed Fakhry Hussein

**Affiliations:** 1 Tropical Health Department, High Institute of Public Health, Alexandria University, Alexandria, 21561 Egypt; 2 Department of Pathology, Microbiology and Forensic Medicine, School of Medicine, The University of Jordan, Amman, 11942 Jordan; 3 Department of Clinical Laboratories and Forensic Medicine, Jordan University Hospital, Amman, 11942 Jordan; 4 Department of Translational Medicine, Faculty of Medicine, Lund University, 22184 Malmö, Sweden; 5 Internal Medicine Department, Faculty of Medicine, Alexandria University, Alexandria, 21526 Egypt; 6 Clinical Research Administration, Alexandria Directorate of Health Affairs, Alexandria, Egypt; 7 Ministry of Health and Population, Cairo, Egypt; 8 Occupational Health and Industrial Medicine Department, High Institute of Public Health, Alexandria University, Alexandria, 21561 Egypt

**Keywords:** COVID-19 vaccine, Influenza vaccine, Booster dose acceptance, Vaccine hesitancy, Libya

## Abstract

**Background:**

Coronavirus disease 2019 (COVID-19) vaccine coverage remains low in Libya compared to other countries in the Eastern Mediterranean Region. This study aimed to evaluate the willingness of the general public in Libya to receive COVID-19 and seasonal influenza vaccines. Additionally, the study aimed to investigate the potential effect of combining the two vaccines to reduce COVID-19 vaccine rejection.

**Methods:**

An anonymous nationwide online cross-sectional survey was carried out from 1st September to 16th October 2022. Libyans aged 18 years or older were recruited using convenience and snowball sampling approaches. The participants were surveyed for sociodemographic information, health status, and vaccination attitude towards COVID-19 and seasonal influenza vaccines.

**Results:**

A total of 2484 participants formed the final study sample: 68.7% were females, 39.4% were aged 18–25 years, 50.4% were single, 32.5% had previous COVID-19 infection, and 47.2% experienced COVID-19 death among relatives. Three-fourths of the respondents showed COVID-19 vaccine rejection: 57.3% did not receive COVID-19 vaccination, 10.1% would not complete the primary vaccination series, and 7.8% refused booster doses. About 55.0% rejected seasonal influenza vaccination, while 1.9% reported influenza vaccine uptake and 21.2% were willing to get the influenza vaccine for the first time. Additionally, 18.8% had already received influenza vaccination in the last year and intended to get the vaccine this season, while 3.3% were unwilling to get influenza vaccination this year despite receiving it in the last influenza season. Age, sex, and occupation were significantly associated with COVID-19 and influenza vaccine rejection. Rejection of COVID-19 vaccination decreased if its combination with influenza vaccine as a single dose was suggested, with 28.2% of the COVID-19 vaccine rejector group accepting the combined vaccine as it would be safer (50.9%), needing fewer injections (24.0%), would be more effective (19.1%), and would be less expensive (3%). Approximately 73.0% of the COVID-19 vaccine rejector group refused this combination due to fear of side effects (48.7%), absence of published studies on this combination (29.8%), and considering this combination as useless (11.2%).

**Conclusion:**

In Libya, the prevalence of COVID-19 vaccine rejection was high, while the rejection of seasonal influenza vaccination was relatively lower. If influenza and COVID-19 vaccines are administered simultaneously as a single injection, this may reduce the rejection of the COVID-19 vaccine due to better-perceived vaccine safety and efficacy besides being more convenient in terms of the number of injections and cost.

## Introduction

On 11 March 2020, the World Health Organization (WHO) declared coronavirus disease 2019 (COVID-19), a severe acute respiratory disease, to be a pandemic. Since then, it has grown to be a serious public health issue worldwide with varying patterns of incidence and mortality [1, 2]. The WHO reported around 762.8 million confirmed COVID-19 cases as of 12 April 2023, including 6.9 million fatalities. Since the beginning of the pandemic, Libya has reported 507,229 COVID-19 cases with 6437 fatalities (case fatality ratio of 1.27) [3]. In the Eastern Mediterranean Region (EMR) and towards the end of 2020, it was noteworthy that, Libya had one of the highest rates of COVID-19 cases and fatalities (1405 cases per 100,000 people) and (20 deaths per 100,000 people), respectively [4].

It has been demonstrated that vaccination can considerably reduce severe and deadly COVID-19 cases. The incidence of COVID-19, whether it was symptomatic or asymptomatic, significantly declined following single or two doses of COVID-19 vaccination [5, 6]. The WHO is dedicated to expanding access to COVID-19 vaccines and aiding governments in speeding up vaccine supply to reduce COVID-19-associated morbidity and mortality. Globally, a total of 13,340 billion COVID-19 vaccine doses have been administered till 12 April 2023 as an attempt to reduce the disease incidence and to avoid relying solely on the herd immunity [3]. Libya started the COVID-19 vaccination campaign on 11 April 2021. Up to 28 January 2023, ten vaccine types are being used, with a cumulative number of 3.7 million COVID-19 vaccine doses that were administered to a total of 2.3 million Libyans. The total number of vaccine doses administered per 100 population was 54.4, with 33.7% who received at least a single dose of COVID-19 vaccination, and only 2.7% who received the booster or additional doses [7].

Seasonal influenza, commonly known as the flu, is an acute, highly contagious viral infectious disease that spreads through respiratory droplets [8]. The WHO estimates that 1.5 billion people globally contract seasonal influenza each year, with 3–5 million developing severe diseases that may necessitate hospitalization and result in death. Furthermore, an estimated 650,000 people die each year as a result of influenza infection [9]. The WHO emphasizes that the primary method of preventing seasonal influenza is through yearly vaccination [8]. The general population should receive annual vaccination with seasonal influenza vaccines before the start of the influenza season, with a particular focus on high-risk groups. This strategy can significantly reduce influenza-related morbidity, mortality, and economic losses [10, 11]. In addition to the vaccine's direct benefits, decreased viral circulation can also have an indirect beneficial impact on other community members. Public health organizations have advised maintaining or boosting influenza vaccination rates is essential during the ongoing COVID-19 pandemic. This is to reduce the impact of seasonal influenza on the healthcare systems that are already overburdened [12]. However, following vaccination, approximately 2–10% of healthy individuals who have been vaccinated may fail to develop sufficient antibodies [13]. This inappropriate response may be due to factors such as the individual's immune system status or genetic characteristics, such as the type of human leukocyte antigen (HLA) or single-nucleotide polymorphisms [14].

Because influenza and COVID-19 are both caused by respiratory viruses with similar modes of transmission, there is a growing concern among researchers that these two viruses may coexist during the winter and spring seasons in the upcoming years [15]. As a result, there may be an increase in demand for healthcare services, higher utilization of limited medical resources, and added burden on public health. In this scenario, influenza vaccination is necessary not only to avoid co-infection but also to minimize the number of flu patients, freeing up space for COVID-19 patients in healthcare facilities. According to early studies, COVID-19 patients who tested positive for influenza IgM had a lower risk of mortality or were less likely to suffer from severe COVID-19 disease compared to influenza IgM-negative patients [16]. Moreover, a lower incidence of COVID-19 infection has been linked to influenza vaccination. In a study conducted by Conlon et al. [17] patients who got an influenza vaccination had a lower risk of testing positive for COVID-19 compared to those who did not receive the vaccination. Studies have shown that vaccinated patients who tested positive for COVID-19 were less likely to require hospitalization or mechanical ventilation and had a shorter hospital length of stay compared to unvaccinated individuals [17–19]. Furthermore, vaccinated individuals had lower odds of COVID-19-related mortality [20, 21].

Of note, seasonal influenza vaccines are widely accessible; however, influenza vaccine rejection is relatively common [22]. Recent studies have indicated a decline in seasonal flu vaccination rates during the COVID-19 pandemic in 2020, despite a marvelous increase in vaccination rates from 2017 to 2019 [23, 24]. Moreover, there was a significant number of healthcare workers (HCWs) who are hesitant to receive seasonal influenza vaccination [25].

Evidence-based planning is required to increase the uptake of both vaccines before their introduction since there is a significant proportion of hesitancy towards both influenza and COVID-19 vaccination. Through an analysis of the causes of the shift in vaccine acceptability for influenza, the actual patterns of vaccination acceptance for COVID-19, and the efficacy of promotion tactics, policymakers can develop a comprehensive strategy and specific public health measures to expand vaccine coverage further. This study aimed to investigate the attitude of the general public in Libya towards COVID-19 and influenza vaccination, as well as the possible factors associated with vaccine rejection. Moreover, we aimed to investigate the impact of combining COVID-19 vaccine with the seasonal influenza vaccine on reducing COVID-19 vaccine rejection.

## Materials and Methods

### Study Setting

We conducted this nationwide cross-sectional study in Libya between September 1 and October 16, 2022. To collect data, an electronic anonymous online survey was created using google form and distributed via multiple social media and messaging platforms, including Facebook, Messenger, WhatsApp, and Instagram. In addition to these platforms, the survey was also sent out through email.

### Study Population and Sampling Methods

The study used convenience and snowball sampling approaches to recruit participants. The target participants fulfilled the following inclusion criteria: (1) living in Libya during the time of the survey, (2) being 18 years or older, (3) having a smart phone or computer with access to the Internet, (4) be educated to self-complete the survey.

### Sample Size

The study determined that a sample size of 566 participants would be sufficient to achieve a 5% margin of error, a 95% confidence level, and a 60% response rate, given an estimated rate of COVID-19 non-vaccinated population of 67% [26]. However, to account for stratification during data analysis including the presence of two governments in Libya; Hukumat al Wahdat based in Tripoli and Al-Thani Cabinet, based in Tobruk and influenza vaccination status (vaccinated vs not vaccinated)], the sample size was multiplied by four to be 2226.

(N) = ([Z^2^ × P × (1 – P)/(E)2] × Deff × authorities in Libya)/expected response rate. N = (1.9622*0.67(1–0.67)/(0.05)2*2*2)/0.60 [27].where:

Z—st. normal distribution = 1.96;

P—Proportion = 67%;

N—Total Libyan Population = 7.085 million;

E—A margin of error = 0.05% (with 95% confidence interval).

### Tools of Data Collection

A questionnaire of four sections was created to collect the data. The first section collected the sociodemographic and health-related condition data and included question about age, sex, education, marital status, working status, history of chronic disease (heart disease, type 2 diabetes mellitus (DM), stroke, cancer, obesity, and arthritis), previous COVID-19 infection, and COVID-19 among relatives). The questionnaire’s second section focused on the attitude toward the COVID-19 vaccine (I took the first, second, and booster doses, I waited for the booster dose, I waited for the second dose, I would not take the booster dose, I took the first dose but would not take any other doses, and I would not take any doses). The third section of the questionnaire collected data on the attitude toward the seasonal influenza vaccine (I took the vaccination last year and current year, I took vaccination last year and I was waiting for the vaccine this year, did not get vaccinated before but would take it this year, I took it last year but would not take it this year, I did not take the vaccination before and I would not take it this year). The fourth section of the questionnaire was designed to gather data on the attitude of the participants who had not yet received the COVID-19 vaccine. Specifically, they were asked about their willingness to receive the vaccine if it is combined in a single shot with the seasonal influenza vaccine. Then, participants who accepted or rejected this hypothetical combination were asked about causes of such decision.

### Plan of Data Collection

Prior to data collection, the research team conducted a pilot study to assess the feasibility and accessibility of the online tool. Collaborator were required to provide at least two responses and give their feedback to improve the flow and clarity of the study questionnaire. Based on the piloted study feedback some minor edits were made. Furthermore, the pilot study helped us to determine the expected response rate and time required to complete the survey.

### Ethical Considerations and Approval

The study was approved by The Ethics Committee of the Faculty of Medicine, Alexandria University, Egypt (IRB number: 00012098). The study was conducted in accordance with the ethical standards outlined in the 1964 Declaration of Helsinki and its later amendments or comparable ethical standards [28]. All participants were informed that their participations were voluntary, and a consent was obtained by answering a question prior to starting the survey (“to agree” or “not to agree”) to participate in the study. Participants did not receive any incentive in return for their participation. Responses were saved in a password-protected computer accessible only to the lead investigator to ensure data confidentiality.

### Statistical Analysis

The R 4.2.1 program (R Foundation for Statistical Computing, Vienna, Austria) was used to manage and analyze data. Figures were created using Microsoft Power Point and the ggplot2 package. Responders were classified as vaccine acceptors (those received the 1st dose and waiting the second, received second dose of vaccination and waiting booster, or already received the booster dose) or vaccine rejectors (those who refused to receive any dose of vaccination, those receive the first dose and refused to complete the primary series of vaccination, and those who refused to receive booster dose of vaccination). Numerical variables were described by their mean and standard deviation (SD), whereas categorical variables were described by the number and percentage (%). A Chi-Square test was used to assess the association between the categorical variables, and the responses were categorized according to COVID-19 or influenza vaccinations status. An independent t-test was performed to compare the difference between means of the two independent groups. A *p*-value < 0.05 was considered statistically significant.

## Results

### Respondents’ Sociodemographic Characteristics

A total of 2484 participants responded to this survey, with 69 being excluded as their age was below 18 years. Nearly 68.7% (1660) of the participants were females, 39.3% (950) were aged 25–35 years old, 50.4% (1216) were single, 59.6% (1440) had a university degree, 10.4% (252) had chronic diseases, around 32.5% (785) had previously contracted COVID-19 infection, and 47.3% (1141) had lost relatives due to COVID-19 (Table 1).

**Table 1:** Sociodemographic criteria and medical conditions, n = 2415

Variables (n = 2415)	n (%)
Sex	
Males	755 (31.3)
Females	1660 (68.7)
Age	
18–25 years	832 (34.7)
25–35 years	950 (39.5)
35–50 years	566 (23.5)
50–65 years	67 (0.3)
Marital status	
Single	1216 (50.4)
Married	1108 (45.6)
Divorced	91 (3.8)
Education	
Primary Education	14 (0.6)
Secondary education	557 (23.1)
University degree	1440 (59.6)
Postgraduate	404 (16.7)
Working	
I do not work	186 (12.7)
Retired	50 (3.4)
Students	293 (20.0)
Working in the medical field	526 (35.8)
Working outside the medical field	413 (28.1)
Chronic disease	
Yes	252 (10.4)
No	2163 (89.6)
Previous COVID-19 infection	
Yes	785 (32.5)
No	1134 (47.0)
Do not know	496 (20.5)
A relative died due to COVID-19 infection	
Yes	1141 (47.3)
No	1160 (48.0)
Do not know	114 (4.7)

### Attitude of Respondents Towards COVID-19 and Influenza Vaccines

**Fig. 1:**
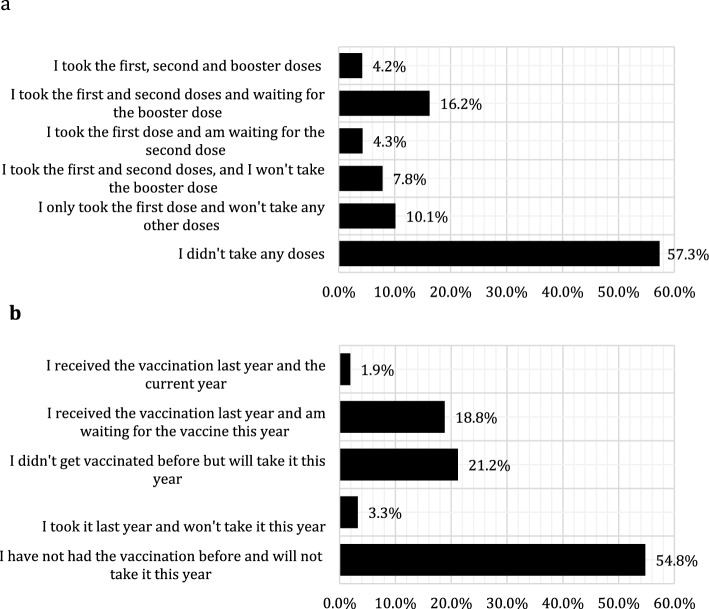
**a** Acceptance of COVID-19 vaccination among the Libyan participants, **b** acceptance of seasonal influenza vaccine among Libyan participants

Nearly two-thirds 57.3% (1385) of the respondents were rejecting COVID-19 vaccination, 10.1% (245) received the first dose and would not complete the vaccination schedule, and 7.8% (189) were fully vaccinated and refused the booster doses. While 4.3% (103) received the first dose of COVID-19 vaccination and were waiting for the second dose, 16.2% (392) had received the two doses and were waiting for the booster dose, and 4.2% (103) had already received the booster dose (Fig. 1a). About 54.8% (1323) of the participants were rejecting seasonal influenza vaccination, and 3.3% (79) had already received it in the last year but they were unwilling to receive the current seasonal influenza vaccine. About one-fifth 21.2% (512) planned to get the seasonal influenza vaccine for the first time, 18.8% (455) had received vaccination in the last year and intended to get the vaccine in current influenzas season, and 1.9% (46) had already received the current influenza season vaccine (Fig. 1b).

**Fig. 2:**
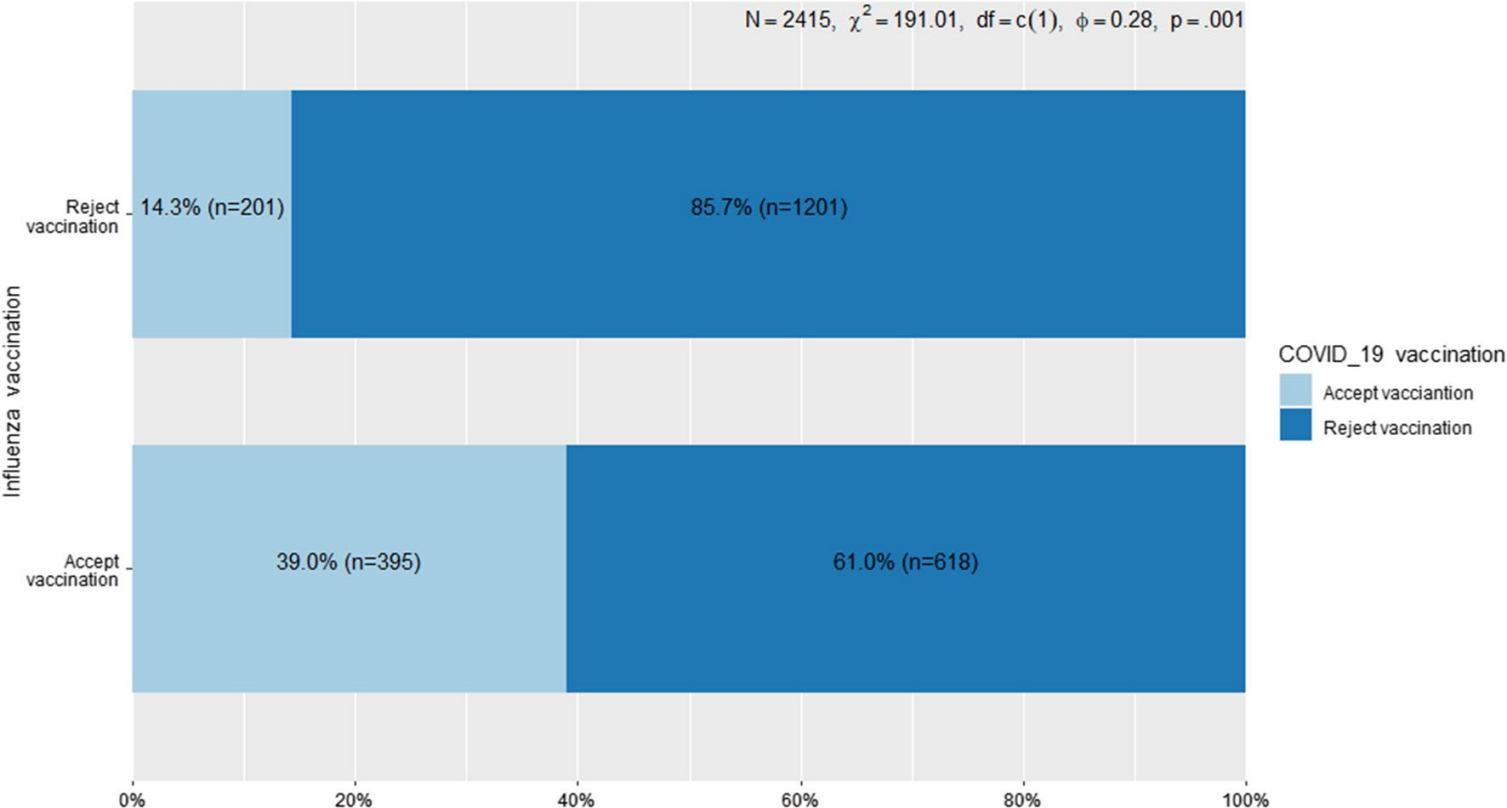
Association between attitude towards COVID-19 vaccine and attitude towards influenza vaccine among the respondents. The blue color indicates COVID-19 rejection across the attitude towards seasonal influenza vaccine. The cyan color indicates acceptance of COVID-19 vaccination across the attitude towards seasonal influenza vaccination

Figure 2 shows a statistically significant association between attitude towards influenza and COVID-19 vaccination. Nearly, three-fourths 75.3% (1819) of the respondents rejected COVID-19 vaccination (either did not receive any doses, refused to complete the basic immunization, or refused booster doses). About 58.1% of the participants refused the seasonal influenza vaccine (either did not receive the vaccine before or refused to take the current season vaccine). It is worth noting that 66.0% of those who rejected seasonal influenza vaccine  had also rejected COVID-19 vaccination, while 34.0% of those who accepted seasonal influenza vaccine had also accepted COVID-19 vaccination. This difference was statistically significant, χ^2^ = 191.0, df = 1, *p* < 0.0001. The Phi Coefficient was 0.28 indicating moderate positive correlation between rejection of influenza vaccine and rejection of COVID-19 vaccine.

### Characteristics of Vaccinated and Non-vaccinated Participants

Acceptance of COVID-19 vaccination was the highest among the population aged 50–65 years old at 55.2% (37), while it was the lowest among participants aged 25–35 years and 18–25 years at 20.1% and 22.6% respectively. This difference was statistically significant *p* < 0.001. Likewise, the acceptance of influenza vaccination was found to be higher among the population aged 50–65 years (52.2%), and lowest among participants aged 25–35 years (35.6%) and aged 18–25 years (44.8%), with a statistically significant difference (*p* < 0.001). Sex was found to be significantly associated with the acceptance of both COVID-19 and seasonal influenza vaccine; Males were accepting the COVID-19 vaccine more than females (29.8% vs 22.3%, *p* < 0.001). Similarly, males were more accepting seasonal influenza vaccination than females (45.0% vs 40.5%, *p* = 0.042). 29.8% of those who contracted COVID-19 infection accepted vaccination versus 24.1% of those who did not contract COVID-19 infection accepted COVID-19 vaccination. However, this difference was not statistically significant *p* = *0.108*. Finally, occupation was associated with both vaccines’ acceptance. COVID-19 vaccine was highest among clerks (33.3%) and professional jobs (31.3%) while it was lowest among retired and non-working (13.2%) and among craft and related trades workers (17.9%). Regarding seasonal influenza vaccine acceptance, retired and not working people followed by students showed the lowest rates of vaccine acceptance (32.3% and 41.2%) respectively. It is worth noting factors such as marital status, presence of chronic diseases, and having relatives died from COVID-19 did not show a significant association with vaccines’ rejection in Table 2.

**Table 2:** Comparison of COVID-19 and influenza vaccines acceptors and rejectors

Variables	Total (n = 2415)	*COVID-19 vaccination*			*Influenza vaccination*		
		Accepting vaccination n (%)	Refused vaccination n (%)	*p*	Accepting vaccination n (%)	Rejecting vaccination n (%)	*p*
Age							
18 to less than 25 years	832	188 (22.6)	644 (77.4)	< 0.001	373 (44.8)	459 (55.2)	< 0.001
25 to less than 35 years	950	191 (20.1)	759 (79.9)		338 (35.6)	612 (64.4)	
35 to less than 50 years	566	180 (31.8)	386 (68.2)		267 (47.2)	299 (52.8)	
50–65 years	67	37 (55.2)	3 (44.8)		35 (52.2)	32 (47.8)	
Sex							
Female	1660	371 (22.3)	1289 (77.7)	< 0.001	673 (40.5)	987 (59.5)	0.042
Male	755	225 (29.8)	530 (70.2)		340 (45.0)	415 (55.0)	
Marital status							
Divorced	91	24 (26.4)	67 (73.6)	0.929	45 (49.5)	46 (50.5)	0.332
Married	1108	273 (24.6)	835 (75.4)		463 (41.8)	645 (58.2)	
Single	1216	299 (24.6)	917 (75.4)		505 (41.5)	711 (58.5)	
Previous COVID19 infection							
No	2163	521 (24.1)	1642 (75.9)	0.108	902 (41.7)	1261 (58.3)	< 0.001
Yes	252	75 (29.8)	177 (70.2)		111 (44.0)	141 (56.0)	
Do not know	496	105(21.2)	391(78.8)		175(35.3)	321(64.7)	
Relatives died due to COVID19							
No	1134	285 (25.1)	849 (74.9)	0.194	519 (45.8)	615 (54.2)	0.897
Yes	785	206 (26.2)	579 (73.8)		319 (40.6)	466 (59.4)	
Do not know	114	20 (17.5)	94 (82.5)		48 (42.1)	66 (57.9)	
Chronic diseases							
No	1160	290 (25.0)	870 (75.0)	0.057	492 (42.4)	668 (57.6)	0.518
Yes	1141	286 (25.1)	855 (74.9)		473 (41.5)	668 (58.5)	
Occupation							
Army	30	7 (23.3)	23 (76.7)	< 0.001	12 (40.0)	18 (60.0)	0.017
Clerical Support Workers	147	49 (33.3)	98 (66.7)		64 (43.5)	83 (56.5)	
Craft and Related Trades Workers	190	34 (17.9)	156 (82.1)		91 (47.9)	99 (52.1)	
Manager	490	132 (26.9)	358 (73.1)		227 (46.3)	263 (53.7)	
Not working/Retired	303	40 (13.2)	263 (86.8)		98 (32.3)	205 (67.7)	
Professional job as in the medical field or engineer or chemist	527	165 (31.3)	362 (68.7)		223 (42.3)	304 (57.7)	
Service and sales workers	117	22 (18.8)	95 (81.2)		49 (41.9)	68 (58.1)	
Student	587	139 (23.7)	448 (76.3)		242 (41.2)	345 (58.8)	
Others	24	8 (33.3)	16 (66.7)		8 (33.3)	16 (66.7)	

### Determinants of Booster Dose Acceptance

**Fig. 3:**
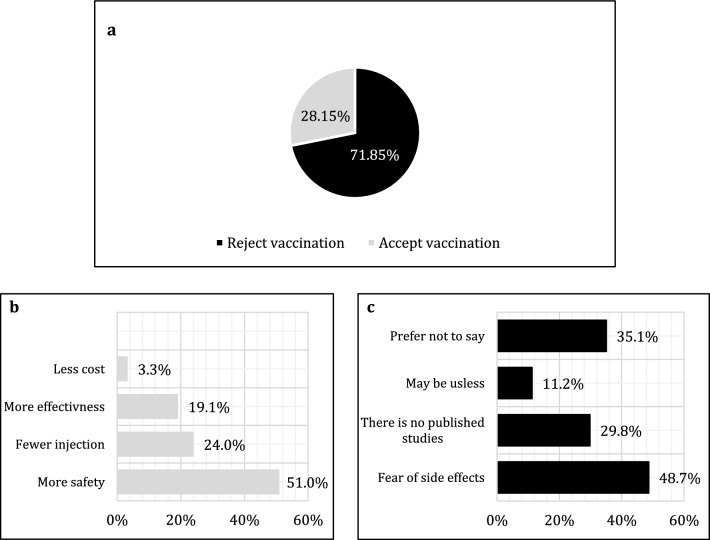
**a** Acceptance of hypothetical vaccines combines COVID-19 and influenza vaccines, **b** causes of acceptance of the hypothetical vaccines combines COVID-19 and influenza vaccines, **c** causes of rejection of the hypothetical vaccines combines COVID-19 and influenza vaccines among Libyan participants

Among the 1819 participants who refused to get COVID-19 vaccination, 28.2% (512) were willing to accept COVID-19 and influenza vaccinations if they were administered in one shot (Fig. 3). These participants accepted the combination as they considered it safer 51.0% (261), has fewer injections 24.0% (123), more effective 19.1% (98), and less expensive 3.3% (17) (Fig. 3). However, 71.85% (1307) rejected this combination due to fear of its side effects 48.7% (529), absence of any published studies that prove the effectiveness of this combination 29.8% (324), and their belief that this combination may be useless. 11.2% (147) (Fig. 3).

## Discussion

According to our study, 57.3% of the Libyans participants reported that they did not receive the COVID-19 vaccines, 10.1% received the first dose and would not complete the primary series of vaccination, and 7.8% had already received the primary series of vaccination and would not take the booster doses. This means that 75.3% of the Libyan participants either did not receive the primary vaccination series or reported that they would not receive booster doses. Interestingly, our study found that rejection rate was lower for the seasonal influenza vaccine. Specifically, 54.8% of the Libyan participants reported rejecting the seasonal influenza vaccine and 3.3% of those who received seasonal influenza vaccine during the last season reported that they would not get vaccinated in the coming influenza season. This means about 58.1% of respondents were rejecting seasonal influenza vaccine. Interestingly, we also found that 66% of those who rejected seasonal influenza vaccine also rejected COVID-19 vaccine. Moreover, rejection of the two vaccines was more common among youth, females, and those who previously had COVID-19. Interestingly, we also found that 28.15% of those who were hesitant about receiving COVID-19 vaccine would receive it if administered concurrently with seasonal influenza vaccine. The main reasons for accepting this combination were perceived higher safety, fewer injections, more effectiveness, and lower cost.

### High Rates of Vaccine Rejection

In this study we found that there is a high level of COVID-19 vaccine rejection (75.3%) among the Libyans participants. On the other hand, Buzgeia et al. [29], conducted a cross-sectional study in March 2021 to assess and compare the acceptability of the COVID-19 vaccine among the general public and students in Benghazi. The study utilized a self-administered questionnaire and included 440 respondents, comprising 240 general population and 200 students. Approximately two-thirds of respondents agreed to get vaccinated against COVID-19 when it became available, whereas the majority (71.4%) stated they would wait some time before taking the vaccine. However, we speculate that the sample size may be not representative of the Libyan population as there was no stratification (students and general population) in sample size calculation. Of note, Buzgeia and her colleagues did not report any significant influence of sociodemographic variables on respondents' attitude towards COVID-19 vaccination uptake. In the same vein, higher rejection rate was reported by Abrina et al. [30] in their study, which utilized a cross-sectional quantitative approach employing the stratified random sampling technique. They recruited 2000 people in April 2021 and found that 47.8% of the respondents reported that they would take the vaccination if it was available. Similar to our findings, Abrina et al. [30] reported higher rates of COVID-19 vaccine acceptance among men; however, population aged 18–25 had higher willingness to get the vaccine. Moreover, they found that being married and working at governmental organization were significantly associated with COVID-19 vaccine acceptance. In fact, COVID-19 vaccine rejection has become a main challenge in EMR, especially in countries with low vaccination coverage like Libya, Sudan. Iraq, and Syria. Many studies have reported high rates of vaccine hesitancy and rejection in the region either towards the primary series of vaccination [31–34] or the booster dose [35, 36]. This negative attitude towards COVID-19 vaccination has also affected parents’ willing to vaccinate both healthy and diseased children against COVID-19 [37, 38]. The negative attitude towards COVID-19 vaccination may be due to different reasons such as lack of trust in safety, effectiveness, and efficacy of the vaccine or mistrust in health care system itself [39]. Misinformation and rumors about COVID-19 and its vaccines, often referred to as 'infodemics,' may also be a significant factor in shaping this negative attitude towards vaccination [40].

### Impact of Adding COVID-19 to Seasonal Influenza Vaccine

In this survey, 28.15% of those who rejected COVID-19 vaccine expressed willingness to receive the combination of seasonal influenza and COVID-19 vaccines if they were administered simultaneously. The finding that the community was willing to accept a novel combination vaccination implies that combining new vaccines or boosters (such as COVID-19) with more widely accepted vaccines, such as seasonal influenza vaccine could be a simple effective way to promote adoption of future innovative vaccines. It appears that respondents accepted this combination as they thought it is safer, more effective, has less cost, and with fewer number of injections. Policymakers and stakeholders should consider this finding and explore the possibility of administering the COVID-19 and seasonal influenza vaccines simultaneously in a single injection, given the community's acceptance of such a combination. This should be conducted hand in hand with conducting more research on studying the effectiveness and safety of such combination on the short and long run. This can address concerns and increase confidence in this combination among those who initially refused this idea. In the same vein, from May to June 2021, a nationally representative sample of 12,887 individuals in the United States were surveyed to gather their opinions on COVID-19 booster dose vaccination, seasonal influenza vaccine, and the combined seasonal influenza vaccine and COVID-19 booster vaccinations. The overall acceptability for a single COVID-19 booster dose vaccination was 45%, while for a single seasonal influenza vaccine, it was 58%. The combination of seasonal influenza and COVID-19 booster vaccination had an overall acceptability of 50% [41]. However, it appears that concerns about the COVID-19 vaccine caused some people who normally receive the seasonal influenza vaccination to reject a combination vaccine. To avoid a decrease in influenza vaccination in this population, it may be necessary to provide both the combination vaccine and the seasonal influenza vaccine separately.

### Strengths and Limitations

This study has a number of limitations. First, because of the inherent disadvantages of cross-sectional online surveys, there may be a sampling bias that limits the representativeness of the data. Secondly, there was a possibility of recall bias and social desirability bias, which may lead to distorted self-reported data. Third, research participants were chosen using a non-random sample strategy, which may limit the external validity and generalization of the findings to larger population. Fourth, we did not assess the specific reasons of vaccines’ rejection among the study participants. Fifth, future research should consider studying the same phenomenon while taking into account the age structure of the Libyan population, particularly older adults. In our study, this age group was underrepresented due to various reasons, such as not owning smartphones. In addition, older adults make up only 4.8% of the total population. However, this age group was at a higher risk of COVID-19 and influenza sequelae due to their underlying health conditions. However, this is study is unique in exploring the impact of adding COVID-19 to seasonal influenza vaccine in attempts to increase vaccine acceptance in countries with high rates of vaccine rejection. Nonetheless, more research are needed to addressed different reasons behind COVID-19 vaccine rejection among this population.

## Conclusion

The rejection of COVID-19 vaccines in Libya is concerning, as more than half of the studied population did not receive any doses of the vaccine, and around one quarter were unwilling to complete the initial series of vaccination or receive booster shots. On the other hand, better acceptance was observed towards seasonal influenza vaccine. Acceptance of COVID-19 vaccine can be improved if both vaccines are given simultaneously in one shot, as the studied population thought this might be safer and more effective with minimal costs. However, it seems that concerns about the COVID-19 have led some people who would normally receive the seasonal influenza vaccine to reject a combination vaccine.

## Data Availability

All data are available upon request by emailing the first author.
